# Effect of Alcohol Consumption on Variceal Rebleeding and Mortality

**DOI:** 10.1155/1992/24068

**Published:** 1992

**Authors:** J. Michael Henderson

**Affiliations:** Department of General Surgery The Cleveland Clinic Foundation 9500 Euclid Avenue Cleveland Ohio 44195-5043 USA


### REFERENCES

1. Pagliaro, I., Burroughs, A.K., Sorensen, T.I.A., Lebrec, D., Morabito, A., D'Amico, G. and Tine, F. (1989) Therapeutic controversies and randomised controlled trials: prevention of bleeding and re-bleeding in cirrhosis. *Gastroenterology International* **2**, 71–84
2. North Italian Endoscopic Club for the Study and Treatment of Esophageal Varices (1988) Prediction of the first variceal haemorrhage in patients with cirrhosis of the liver and esophageal varices. *N.Engl.J.Med.*, **319**, 983–989
3. The Veterans Affairs Co-operative Variceal Sclerotherapy Group (1991) Prophylactic sclerotherapy for oesophageal varices in men with alcoholic liver disease. *N.Engl.J.Med.*, **324**, 1779–1784
4. Pagliaro, L., Burroughs, A.K., Sorensen, T.I.A., Lebrec, D., Morabito, A., D'Amico, G. and Tine, F. (1990) Beta-blockers for preventing variceal bleeding. *Lancet*, **336**, 1001–1002

### EFFECT OF ALCOHOL CONSUMPTION ON VARICEAL REBLEEDING AND MORTALITY

### ABSTRACT

*McCormick, P.A., Morgan, M.Y., Phillips, A., Yin, T.P., McIntyre, N. and Burroughs, A.K. (1992) The effects of alcohol use on rebleeding and mortality in patients with alcoholic cirrhosis following variceal haemorrhage. Journal of Hepatology;* 14, *99–103.*

**The effect of continued alcohol intake on prognosis in alcoholic cirrhotics who have already bled from varices is controversial. To investigate the effect of alcohol intake on prognosis we studied 189 consecutive alcoholic cirrhotics admitted, for the first time, to the Royal Free Hospital with variceal bleeding. Sixty-six died within 30 days of admission and 23 were excluded from the study for other reasons. Of the 100 remaining 15 remained 'probably abstinent' over long-term follow-up, 29 drank occasionally and 56 continued to misuse/abuse alcohol. The percentage survival probability at 2 years was 66% in the probable abstainers, 68% in the occasional drinkers and 63% in the alcohol abuse/misuse group. There were no significant differences in either mortality or rebleeding rates between the three groups. A rebleeding index (designed to take account of the number of rebleeds per patient and the total length of follow-up) also failed to show any significant difference between the three groups. The Cox proportional hazard model was used to study the effect of the following factors on rebleeding and mortality; age, sex, alcohol use, Pugh's score, acute treatment received for initial variceal bleed and long-term treatment received for prevention of recurrent variceal haemorrhage. Pugh's score was significantly related to risk of death during follow-up ($p$ = 0.0122), but none of the others factors was significantly related to risk of rebleeding or mortality. Using conventional methods to determine alcohol use we were unable to demonstrate significant effects of alcohol intake on rebleeding or mortality in alcoholic cirrhotics who had bled from oesophageal varices.**

**PAPER DISCUSSION**

KEY WORDS: Alcohol, alcoholic cirrhosis, oesophageal varices

The abstract cited above shows, somewhat surprisingly, that continued alcohol use/abuse does not have a detrimental effect on survival or rebleeding rate following an initial variceal bleed. Before telling our patients that continued drinking is not bad, let us examine the evidence a little further.

The current paper has some unusual features which may limit the general applicability of these data. First, the particular interest of this excellent Hepatology group in the longterm management of patients with alcoholic liver disease may have influenced their findings. Presumably, most of the patients in this study had their first physician contact at the time of their initial bleed. What changed thereafter? While the assessment of continued drinking in this study is probably as good as can be achieved, the authors do not provide objective evidence of what effect this had on the liver. Table 3 gives mixed messages, with good serum albumin levels on one hand, and elevated bilirubin levels and ascites and encephalopathy in half the patients on the other hand. This could be interpreted as indicating an overall improvement in nutritional status despite ongoing active liver disease. Was nutrition emphasized as an important treatment factor at follow-up? And, if so, might such recommendations account for their findings? Objective evidence of the presence of alcoholic hepatitis at initial presentation or during subsequent follow-up would provide more persuasive evidence that continued alcoholic use played some role in the end-points evaluated.

A second unusual feature of this study is the lack of definitive management for variceal bleeding in 75% of patients. From the point of view of this study, this does indeed permit assessment of the effect of continued drinking on rebleeding and survival in the *untreated* patient. As discussed below, this may be different to the effect of continued drinking on patients who have received definitive therapy. The rebleeding rates of 50% and 70% at one and two years reaffirm old data[1], and redefine the need to institute some therapy to reduce the risk of recurrent variceal bleeding after an initial bleed. While many of the deaths in the reported study were "liver related", the authors have not defined how many could be directly attributed to rebleeding.

Where do these data fit relative to other studies? The authors address the lack of data on this topic in the literature, and highlight for the reader the difficulty in collecting such data. Their findings are consistent with the only other study which has directly addressed this question[2]. What effect does continued alcohol misuse have on outcome after more definitive therapy? In our own particular population of interest, patients receiving distal splenorenal shunt for prevention of recurrent variceal bleeding, the alcohol patient does behave differently. Poorer survival[3,4], and increased loss of portal perfusion to the liver[4,5], have been shown in this population compared to patients with nonalcoholic cirrhosis. An association between loss of portal flow and active alcoholism has been documented[6], and is supported by improved outcome in patients with prolonged hospitalization prior to surgery, which is presumably associated with improved liver status[7]. As always, the questions remain open as to how good is the documentation of alcohol abstinence, consumption and how far continued drinking accounts for these findings.

In the Emory prospective randomized trial comparing endoscopic sclerotherapy to distal splenorenal shunt[8], it was again the patients with alcoholic liver disease who behaved very differently with the two therapies. Improved survival in patients randomized to sclerotherapy (one third of whom required surgical rescue), was only seen in patients with alcoholic cirrhosis ($p < .01$), and was associated with an initial improvement in quantitative liver function. The increased mortality from liver failure associated with return to drinking in the distal shunt patients in this study, raised the question as to whether the liver is more susceptible to injury from alcohol after such an operative procedure. An argument can certainly be built for altered metabolism, but remains unproven.

We must acknowledge that there are still many interactions of alcohol with the liver which we do not understand. The present data do, however, help clarify two things: first, the risk of rebleeding after an initial variceal bleed is *not* related to the amount of alcohol consumed, and second, that once an alcoholic has bled from varices it is factors other than alcohol consumption which determine survival when no other definitive therapy is instituted. With definitive therapy such as distal splenorenal shunt, continued alcohol abuse is associated with increased mortality. Patients . . . we should continue to advise abstinence.

J Michael Henderson
Department of General Surgery
The Cleveland Clinic Foundation
9500 Euclid Avenue
Cleveland, Ohio 44195-5043
United States of America
2025.34 00 000000333 0127 00001

### REFERENCES

1. Graham, D. and Smith, J.L. (1981) The course of patients after variceal hemorrhage. *Gastroenterology*, **80**, 800–809
2. Powell, W.J. and Klatskin, G. (1968) Duration of survival in patients with Laennec's cirrhosis: influence of alcohol withdrawal, and possible effects of recent changes in general management of the disease. *Am.J.Med.*, **44**, 406–420
3. Zeppa, R., Hensley, G.T., Levi, Ju *et al.* (1978) The comparative survival of alcoholics versus nonalcoholics after distal splenorenal shunt. *Ann.Surg.*, **187**, 510
4. Warren, W.D., Millikan, W.J., Henderson, J.M. *et al.* (1982) Ten years of portal hypertension surgery at Emory: results and new perspectives. *Ann.Surg.*, **195**, 530–542
5. Henderson, J.M., Gong-Liang, J., Galloway, J.R. *et al.* (1985) Portaprival collaterals after distal splenorenal shunt: incidence, magnitude and associated portal perfusion changes. *J. of Hepatol.*, **1**, 649–661
6. Kawasaki, S., Henderson, J.M., Hertzler, G. and Galloway, J.R (1991) The role of continued drinking in loss of portal perfusion after distal splenorenal shunt. *Gastroenterology*, **100**, 799–804
7. Myburgh, J.A. (1990) Selective shunts: the Johannesburg experience. *Am.J.Surg.*, **160**, 67–74
8. Henderson, J.M., Kutner, M.H., Millikan, W.J. *et al.* (1990) Endoscopic variceal sclerosis compared with distal splenorenal shunt to prevent recurrent variceal bleeding in cirrhosis. *Ann.Int.Med.*, **112**, 262–269

